# Synaptic Competition Sculpts the Development of GABAergic Axo-Dendritic but Not Perisomatic Synapses

**DOI:** 10.1371/journal.pone.0056311

**Published:** 2013-02-14

**Authors:** Elena Frola, Annarita Patrizi, Thomas Goetz, Lucian Medrihan, Enrica Maria Petrini, Andrea Barberis, Peer Wulff, William Wisden, Marco Sassoè-Pognetto

**Affiliations:** 1 Department of Neurosciences, University of Turin, and National Institute of Neuroscience-Italy, Torino, Italy; 2 Institute of Medical Sciences, University of Aberdeen, Foresterhill, Aberdeen, United Kingdom; 3 Department of Neuroscience and Brain Technologies, The Italian Institute of Technology, Genova, Italy; University of Nebraska Medical Center, United States of America

## Abstract

The neurotransmitter GABA regulates many aspects of inhibitory synapse development. We tested the hypothesis that GABA_A_ receptors (GABA_A_Rs) work together with the synaptic adhesion molecule neuroligin 2 (NL2) to regulate synapse formation in different subcellular compartments. We investigated mice (“γ2 knockdown mice”) with an engineered allele of the GABA_A_R γ2 subunit gene which produced a mosaic expression of synaptic GABA_A_Rs in neighboring neurons, causing a strong imbalance in synaptic inhibition. Deletion of the γ2 subunit did not abolish synapse formation or the targeting of NL2 to distinct types of perisomatic and axo-dendritic contacts. Thus synaptic localization of NL2 does not require synaptic GABA_A_Rs. However, loss of the γ2 subunit caused a selective decrease in the number of axo-dendritic synapses on cerebellar Purkinje cells and cortical pyramidal neurons, whereas perisomatic synapses were not significantly affected. Notably, γ2-positive cells had increased axo-dendritic innervation compared with both γ2-negative and wild-type counterparts. Moreover heterologous synapses on spines, that are found after total deletion of GABA_A_Rs from all Purkinje cells, were rare in cerebella of γ2 knockdown mice. These findings reveal a selective role of γ2 subunit-containing GABA_A_Rs in regulating synapse development in distinct subcellular compartments, and support the hypothesis that the refinement of axo-dendritic synapses is regulated by activity-dependent competition between neighboring neurons.

## Introduction

During development neurotransmission regulates synapse formation and guides the selective assembly of circuitry. Activity mediates competition between converging inputs, through which more active synapses are stabilized and less active synapses are eliminated [1]–[4]. For instance, in the cerebellum, an imbalance in synaptic activity removes surplus climbing fibers innervating individual Purkinje cells (PCs) [5]–[7]. However, central synapses differ highly in their structural and molecular organization [8], and it is unknown if synapse competition is a general feature of CNS development.

In brain circuits, synapse heterogeneity is exemplified by the numerous types of GABAergic synapses that target distinct subcellular domains (somatic, dendritic or axonal) of principal neurons [9]–[11]. How these selective connections are generated during brain development and how their number is controlled is only partially understood [12], [13]. GABA signaling itself coordinates inhibitory synapse development and activity-dependent regulation of synapse density in neuronal compartments [14], [15]. In one study, reducing GABA synthesis in neocortical interneurons resulted in deficits in perisomatic synapse formation around pyramidal cells [16]. Conversely, loss of GABA_A_ receptors (GABA_A_Rs) from cerebellar PCs in GABA_A_R α1 knockout mice affected axo-dendritic synapses made by stellate cells, but not perisomatic synapses established by basket cells [17]. The interpretation of this result was complicated, however, because PCs express transiently α3-GABA_A_Rs at a time when perisomatic synapses form [18]. Nevertheless, these findings imply that GABAergic activity has a selective effect on inhibitory synapse formation in separate types of neuron and/or different neuronal compartments.

To establish the importance of GABAergic signaling for synapse formation in different neuronal populations, synapse organization could be examined in genetically modified neurons that have reduced sensitivity to GABA. Ideally, to study synapse development *in vivo* neurotransmission should be silenced in only a subset of neurons, in order to directly compare the effects on synapse formation with neighbouring neurons that show intact GABA signaling. Moreover, mutations should not compromise animal survival during the postnatal period of intense synaptogenesis. For example, mice with total knockout of the GABA_A_R γ2 subunit gene die in the first postnatal week [19], making it impossible to study how GABA_A_Rs influence later brain development. Here, we describe a new mouse line, GABA_A_R γ2 knockdown (γ2 KD), that has a strongly reduced expression of the γ2 gene throughout the brain during development. Despite this, γ2 KD mice survive until their third postnatal week, thus covering postnatal synaptogenesis. Remarkably, brains of γ2 KD mice have a mosaic expression of the γ2 subunit gene, resulting in a strong imbalance of GABAergic activity in neighbouring neurons. Thus this mouse line allowed us to study the role of GABA in postnatal brain development and synaptogenesis.

## Materials and Methods

### Generation of γ2 KD mice

**Figure 1:**
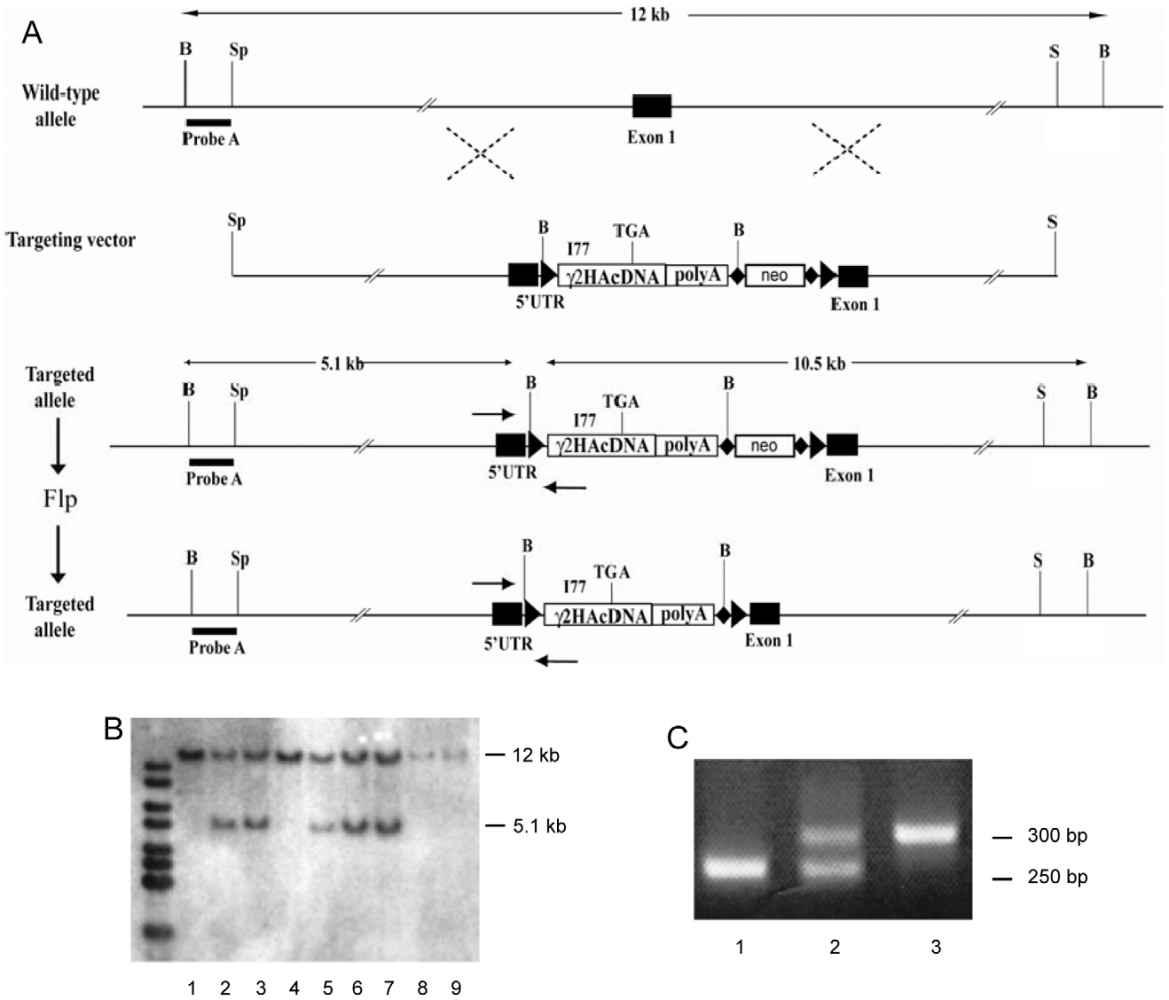
Generation of the γ2F77/I77 gene switch mouse version (γ2 knockdown mouse). **A,** Targeting strategy. A full length γ2 cDNA was placed into the 5′UTR of exon 1 of the GABA_A_R γ2 subunit gene. The γ2 cDNA encodes the I77 version of the γ2 subunit. The 5′loxP contains a BglII restriction site. For the initial targeting a frt-flanked neomycin resistance gene (neo) was placed after the γ2 cDNA. The neo cassette contains a second loxP site at the end, the 3′loxP site. The 5′frt site contains another BglII site. Black bars indicate the positions of Probe A and B. Arrows are PCR primer positions. Black triangle, loxP sites; black diamonds, frt sites; B, BglII site; S, SalI site; Sp, SpeI site; TGA, STOP codon; UTR, untranslated region; HA, hemagglutinin epitope; polyA, polyadenylation signal. **B,** Southern blot analysis of BglII digested tail biopsies hybridized with probe A. Lane 1, 4, 8 and 9 are samples from WT mice. Lane 2, 3 and 5–7 show samples from heterozygous targeted mice. The 5.1 kb band represents the 5′ BglII fragment. **C,** PCR analysis of mouse tail DNA. Lane 1 shows a WT genotype, lane 2 a heterozygous and lane 3 a homozygous (for the mutation) genotype. The PCR amplification is across the 5′loxP site.

The targeting vector was designed such that, by gene targeting in embryonic stem cells, the native GABA_A_R γ2 subunit gene (*gabrg2*, gene reference number ENSMUSG00000020436; www.ensembl.org/Mus_musculus/geneview) had an insertion of a modified γ2 cDNA, flanked by loxP sites, in exon1 (Fig. 1A). The genomic DNA containing the mouse γ2 subunit gene was obtained on a Bacterial Artificial Chromosome (BAC) by screening a mouse 129 BAC library (BAC Mouse ES release I, BAC4921, Genome Systems Inc, USA). As the basis for the targeting vector, an approximately 9 kb *Spe*I/*Sal*I fragment containing exon 1 was subcloned into the *Bam*HI site of pBluescript (Stratagene). Into this 9 Kb fragment, a *Sal*I site was placed by *in vitro* mutagenesis into the 5′UTR region of exon 1, 177 bp 5′ (upstream) of the start-of-translation-ATG codon [20]. Into this SalI site, we placed a cassette containing an HA-epitope tagged γ2 I77 subunit cDNA and SV40 polyadenylation sequence, followed by an frt-flanked neomycin resistance gene (Fig. 1A) [21]. The entire γ2-neomycin cassette was flanked by loxP sites (Fig. 1A). The targeting vector was linearized with Not I, and electroporated into mouse R1 embryonic stem cells (strain 129/Sv). About 800 G418-resistant (Geneticin) ES cell colonies were screened for homologous targeting by Southern blot analysis. Three ES cell colonies had a homologous targeting event. One clone was expanded and the frt-flanked neomycin resistance cassette was removed through transient expression (electroporation) of enhanced FLP (eFLP) recombinase. After confirmation of removal of the neomycin resistance gene (by hybridizing genome DNA of Flp-transfected colonies with a neomycin probe and looking for negative lanes), the targeted ES cells were microinjected into C57BL/6 blastocysts to generate chimeras (by Dr. Frank Zimmermann, University of Heidelberg, Germany). After generation of F1 mice, the genotyping was done by PCR across the 5′loxP site.

The mice used in the present study were generated from heterozygous breeding pairs at our respective institutions. The animals were genotyped by PCR analyses (Fig. 1C) of genomic DNA tail biopsies using the primer pairs:

Pr1 5′-CTG CTT CTC TCA TTT GCC TTC CTG TGT ACA TCT CTG-3′

Pr2 5′-GCT GAT GAT TTG ATG CCG GCT CCC CCC ACC TGC CTC-3′

All procedures for generation and maintenance of mouse lines were done in accordance with the United Kingdom Animals (Scientific Procedures) Act 1986 (Home Office Licence number PPL 60/3562), had ethical approval from the Tierschutz Commission of the Regierungspraesidium Karlsruhe, Germany (project title “veraenderte Ionenkanaele im Gehirn”, granted 30.09.2002), and were approved by the Italian Ministry of Health and by the Bioethic Committee of Turin University in accordance with national (Legislative Decree 116/92 and law n. 413/1993) and international (Directive 86/609/EEC and the recommendation 2007/526/EC from European community) laws and policies.

### In Situ Hybridization

*In situ* hybridization to mouse brain sections with [^35^S]-labeled oligonucleotide probes was performed as described [22]. Non-perfused brains were removed and frozen on dry ice. Sections (14 µm) were cut on a cryostat, mounted onto poly-L-lysine-coated slides, and dried at room temperature. Sections were fixed in 4% formaldehyde, washed in phosphate-buffered saline (PBS), and dehydrated into 95% ethanol for storage at 4°C. Before hybridization, sections were removed from ethanol and allowed to air dry. Probes (0.3 pmol/µl) were 3′ end labelled using terminal deoxynucleotidyl transferase (Roche Diagnostics, Germany) and a 100∶3 molar ratio of α^35^SdATP (250 µCi/µl; Perkin Elmer, UK) to oligonucleotide. Labelled probe, dissolved in hybridization buffer, was applied to sections. Hybridization buffer contained 50% formamide/4× SSC/10% dextran sulphate (1× SSC: 0.15 M NaCl, 0.015 M Na-citrate). Hybridization was at 42°C overnight. Sections were washed with 1× SSC at room temperature for 5 min, 1× SSC at 65°C for 40 min, 0.1× SSC for 1 min at room temperature, 70% ethanol for 1 min at room temperature before 95% ethanol dehydration. Images were generated from four to six-week exposures to Kodak Biomax MR X-ray film (Eastman Kodak, Rochester, NY). To assess non-specific labeling of the sections, each labeled oligonucleotide was hybridized to brain sections with a 100-fold excess of unlabeled oligonucleotide. Oligonucleotide sequences were:

**GABA_A_–α1**: 5′-GAGGGTCCAGGCCCAAAGATAGTCAGAGAGAC CCCGACTTTTCTT-3′

**GABA_A_–β2**: 5′-GGGAAATGACCAAATCCCAAAGTAGCCCCTTTTC CGGACTCTCCA-3′

**GABA_A_–γ1**: 5′-ATGCAAGGTTCCGTATTCCATGAGTGCTGCAAA CACAAAAATGAA-3′

**GABA_A_–γ2**: 5′-AGGAGAGTAGACTGAGCTTCCAATGCTCCATGTA TTTGGCGAACT-3′

**GABA_A_–γ3**: 5′-AGAGGGTGCTTGAAGGCTTATTCGATCAGGAA TCCATCTTGTTGA-3′

**GABA_A_–δ**: 5′-AGCAGCTGAGAGGGAGAAAAGGACGATGGCGTT CCTCACATCCAT-3′

### Antibody characterization

The primary antibodies used in the present study are listed in Table 1. Polyclonal antibodies against the α1 and γ2 subunits of GABA_A_Rs reveal on Western blots single bands of 50 and 43–48 kDa. This labelling is abolished by competition with the respective antigens [23], [24]. Labelling specificity has also been verified in brain sections of knockout mice lacking the corresponding GABA_A_R subunit [19], [25].

**Table 1:** Primary Antibodies Used.

Antibody	Immunogen	Source, ID Number and Species	Dilution
GABA_A_Rα1	Rat N-terminal peptide, aa. 1–16	H. Mohler and J.-M. Fritschy (University of Zürich, Switzerland). Rabbit polyclonal	1∶5000
GABA_A_Rγ2	Rat N-terminal peptide, aa. 1–29	H. Mohler and J.-M. Fritschy (University of Zürich, Switzerland). Guinea pig polyclonal	1∶2000
α-Dystroglycan clone VIA4-1	Rabbit skeletal muscle membrane preparation	Upstate-Millipore (cat. No. Q14118). Mouse monoclonal	1∶100
Neuroligin2	Rat C-terminal peptide, aa. 750–767	F. Varoqueaux (Max Planck Institute of Experimental Medicine, Göttingen, Germany). Rabbit polyclonal	1∶2000
Car8	Mouse peptide, aa. 33–61	M. Watanabe (Hokkaido University, Sapporo, Japan). Guinea pig polyclonal	1∶500
mGluR1α	Rat peptide, aa. 945–1127	M. Watanabe (Hokkaido University, Sapporo, Japan). Guinea pig polyclonal	1∶500
GAD-6	Affinity-purified GAD from rat brain	Developmental Studies Hybridoma Bank, University of Iowa. Mouse monoclonal	1∶1000
Pan Neurofilament Marker (SMI 311)	Homogenates of saline-perfused rat hypothalamus	Covance (cat. No. SMI-311R). Mouse monoclonal	1∶1000
Calbindin D28k	Purified calbindin D28k from chicken gut	Swant (cat. No. 300). Mouse monoclonal	1∶10000
Calretinin	Recombinant human calretinin	Swant (cat. No. 7699/3H). Rabbit polyclonal	1∶2000
Parvalbumin	Purified parvalbumin from carp muscles	Swant (cat. No. 235). Mouse monoclonal	1∶10000

The rabbit anti-NL2 antiserum recognizes a single band of 105 kDa in Western blots of rat and mouse brain homogenates [26]. Immunolabelling is abolished by preabsorption with the peptide antigen, and no bands are visible in Western blots of NL2 knockout mouse brain homogenates. Moreover, the antiserum does not cross-react with NL1 or NL3 in transfected cells.

The monoclonal antibody against α-dystroglycan (α-DG; clone VIA4-1) recognizes a single band of approximately 156 kDa in Western blots of skeletal muscle lysate [27]. In neurons, mAbVIA4-1 gives a punctate labelling that colocalizes with GABA_A_Rs at postsynaptic specializations and is abolished by genetic deletion of dystroglycan (ref. [28] and unpublished observations).

Antibodies against carbonic anhydrase 8 (Car8), a selective marker of Purkinje cells, recognize a single band of 35 kDa in mouse cerebellar homogenates [18]. When applied to immunofluorescence on parasagittal brain sections, the antibodies strongly label Purkinje cells, and the immunoreactivity is abolished by preabsorption with the immunogen.

Mouse monoclonal anti-calbindin reacts specifically with calbindin (28 kDa) in immunoblots of brain homogenates of different species, and does not cross-react with calretinin or other known calcium-binding proteins [29]. No labelling is visible in brain sections obtained from calbindin D-28k knockout mice [30]. The rabbit anti-calretinin antiserum has been previously characterized by Western blotting of sturgeon brain extracts [31], in which the antiserum recognized a single protein band of the appropriate molecular weight. Mouse monoclonal anti-parvalbumin stains the 45Ca-binding spot of parvalbumin (12 kD and IEF 4.9) in a two-dimensional immunoblot [32]. This antibody has also been characterized extensively by immunohistochemistry [29], [33].

The monoclonal GAD-6 antibody has been characterized via Western blot of rat brain homogenates and found to recognize selectively GAD65 but not GAD67 [34]. Epitope deletion studies have demonstrated that the GAD-6 antibody recognizes an epitope located between amino acids 475 and 571 of the C-terminus of GAD65 [35], [36].

The polyclonal guinea pig anti-mGluR1α antibody recognizes a single band of 145 kDa in Western blots of cerebellar homogenates [37]. Specificity of the immunolabeling has also been verified on samples from mGluR1α null mice [38].

The mouse monoclonal antibody SMI 311 was kindly provided by Dr. Rita Garbelli (Besta Neurological Institute, Milan). This antibody recognizes the 50 and 200 kDa components of non-phosphorylated neurofilaments [39], and has been previously used to label cortical neurons and their dendritic profiles [40], [41].

### Immunofluorescence

For detection of postsynaptic molecules (NL2, GABA_A_Rs, α-DG), postnatal mice (P7–P20) were anesthetized with i.p. ketamine-xylazine 1∶1 (0.1 ml/kg) and decapitated. The brains were excised and cut manually in either sagittal (cerebellum) or coronal (cerebral hemispheres) slabs, that were fixed by immersion in ice-cold formaldehyde (4% in 0.1 M phosphate buffer, PB, pH 7.4) for 20–30 min. Alternatively, mice were perfused with 4% formaldehyde in PB, and their brains were postfixed for 3 hours. Tissue slabs were cryoprotected in sucrose, sectioned with a cryostat, and the sections were collected on gelatin-coated slides. Following a blocking step in normal goat or donkey serum (3% in PBS with 0.5% Triton X-100), the sections were incubated overnight with combinations of two or three primary antibodies. The sections were then washed and incubated with the appropriate secondary antibodies, raised either in goat or in donkey, conjugated to one of the following fluorophores: Alexa 488 (Molecular Probes, Eugene, OR), Alexa 568, or the cyanine-derived Cy3 and Cy5 (Jackson Immunoresearch, West Grove, PA). The sections were rinsed again and coverslipped with Dako fluorescence mounting medium (Dako Italia, Italy).

### Confocal microscopy and data analysis

The sections were analyzed with a laser scanning confocal microscope (Zeiss LSM5 Pascal) using the multichannel acquisition mode to avoid fluorescence crosstalk. Quantitative analyses were performed on a minimum of three mice per group. Synaptic structures were analyzed on images acquired with a ×100 oil-immersion objective (1.4 numerical aperture) at a magnification of 8.1×10^−3^ µm^2^/pixel, and the pinhole set at 1 Airy unit. The images were processed with the image-analysis program Imaris (release 4.2; Bitplane, Zurich, Switzerland). After segmentation, synapse density was quantified with NIH Image J software (http://rsb.info.nih.gov/nih-image) as described in detail previously [42]. The number of perisomatic and axo-dendritic synapses was determined by counting manually synaptic clusters at the surface of PCs labeled for carbonic anhydrase 8 (Car8) or pyramidal neurons labeled with a monoclonal antibody against the pan-neuronal neurofilament marker SMI 311. Pyramidal neurons were identified by their typical morphology (triangular shaped cell body, apical dendrite and multiple basal dendrites). Heterologous contacts between GABAergic axon terminals and PC dendritic spines were quantified in confocal images after immunofluorescence labeling with selective markers [18]. Data are expressed as the number of contacts per surface of GABAergic boutons. Confocal imaging of the cerebellum of GABA_A_R α1 knockout mice was performed in sections used for our previous study [18].

### Electrophysiology

Cerebellar PCs were recorded in acute cerebellar slices obtained from postnatal mice (P15–P18) prepared as previously described [43]. Whole-cell voltage-clamp recordings were performed at room temperature using the Multiclamp 700B/Digidata1440A system (Molecular Devices, Sunnyvale CA, USA) at pipette holding voltage of −70 mV. PCs were visually identified using an upright Olympus BX51WI microscope (Olympus, Japan) equipped with Nomarski optics. Patch pipettes, pulled from borosilicate glass capillaries (Hilgenberg, Malsfeld, Germany), showed 4 to 5 MΩ resistance when filled with high-chloride intracellular recording solution containing (in mM): 126 KCl, 4 NaCl, 1 MgSO_4_, 0.02 CaCl_2_, 0.1 BAPTA, 15 Glucose, 5 HEPES, 3 ATP and 0.1 GTP (pH 7.3 with KOH and osmolarity 290 mosmol l^−1^). Slices were continuously perfused with an extracellular solution containing (in mM): 125 NaCl, 25 NaHCO_3_, 25 glucose, 2.5 KCl, 1.25 NaH2PO_4_, 2 CaCl_2_, and 1 MgCl_2_ (pH 7.4 when bubbled with 95% O_2_-5% CO_2_). Kynurenic Acid (1 mM) was added to the extracellular solution to prevent glutamatergic events. Currents were sampled at 50 kHz, filtered at 3 kHz and stored in a computer hard drive. GABAergic IPSCs were analysed using the Clampfit 10.0 detection module (Molecular Devices, Sunnyvale CA, USA) that exploits a sliding template-based algorithm. The threshold for detection was set at 5 times the standard deviation of the baseline noise. For each recording, averaged traces are obtained from at least 50 synaptic events.

The rise time of GABA-elicited currents was estimated as the time needed for a 10 to 90% increase of the peak current response. The decaying phase of currents was fitted with exponential function in the form:
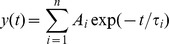
where *Ai* are the fractions of the respective components.

In the case of analysis of normalized currents, Σ*A_i_* = 1. Deactivation time course was fitted with a sum of two exponentials (n = 2) for wild-type (WT) and WT-like cells, and with a monoexponential function (n = 1) for KD cells. Data are expressed as mean ± SEM, and unpaired Student's t-test was used for data comparison.

## Results

### Manipulation of the *gabrg2* gene produces a strong knockdown of its expression

**Figure 2:**
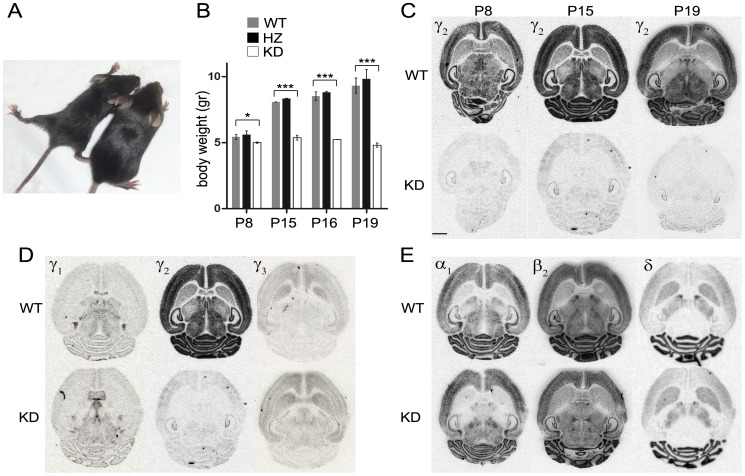
Phenotype of γ2 KD mice and reduced expression of the γ2 subunit gene. **A,** Reduced growth of a P18 γ2 KD mouse (left) compared to a WT littermate. Note also the atypical posture of the mutant mouse. **B,** Bodyweight table of WT, heterozygous (HZ) and homozygous (KD) γ2 KD mice (***, p<0.0001; *, p = 0.0198; unpaired *t*-test; n = 3 mice per group). **C–E,** In situ hybridization on horizontal brain sections from WT and γ2 KD mice. Note the very low expression levels of the γ2 subunit in developing brains homozygous for the γ2 I77 cDNA insertion into the gabrag2 gene (**C**), whereas no obvious change is visible in the expression of the other γ isoforms (**D**) and of other GABA_A_R subunits (**E**). The sections in **D,E** are from P15 mice. Scale bar: 2 mm.

The γ2 KD mouse line arose accidently. We had intended to generate a mouse line with a conditional *gabrg2* allele, provisionally termed “γ2 switch”, that would serve as an elaboration of our method of making subsets of neurons selectively sensitive to zolpidem [44]. By homologous recombination in mouse embryonic stem cells, a γ2 cDNA encoding the zolpidem-insensitive γ2I77 version was placed into the 5′UTR of the native γ2F77 gene followed by a polyadenylation signal (Fig. 1A). In the resulting mice, the endogenous γ2 promoter and regulatory regions were expected to drive the expression of the inserted γ2I77 reading frame in the pattern of the native gene, causing all neurons to be insensitive to zolpidem and β-carbolines [21], [44]. The γ2I77 cDNA was also flanked with loxP sites to allow its removal by Cre recombinase in selected neuronal types, so restoring expression of the original γ2 gene encoding zolpidem-sensitivity. A problem became apparent, however, when we produced homozygous mice for the modified γ2 allele. Mice homozygous for the γ2 subunit switch allele (γ2 KD mice) were normal at birth, but soon developed several abnormalities, including reduced growth (Fig. 2A,B), hunched posture, hyperactivity, impaired grasping and righting reflex, and died around P20. Mutant mice were not obtained at the expected Mendelian frequency, and the breeding of γ2 KD mice was extremely time consuming due to drastically reduced reproduction rates.

We evaluated the expression of the γ2 subunit using *in situ* hybridization on brain sections obtained from postnatal γ2 KD mice, heterozygous and WT littermates. Normally, the γ2 subunit gene is transcribed from before embryonic stage 14, and has a sustained strong expression throughout embryonic and postnatal development in most regions of the CNS [45]. γ2 KD mice had a dramatically reduced level of γ2 transcripts during brain development (Fig. 2C). However, there were no changes in the expression of the γ1 and γ3 subunit genes, suggesting that the γ2 KD phenotype is not compensated by increased expression of either the GABA_A_R γ1 or GABA_A_R γ3 subunits (Fig. 2D). Similarly, there were no changes in mRNA hybridization signals for other major GABA_A_R subunits, such as α1, β2 and δ (Fig. 2E). As the γ2 subunit is required for the postsynaptic accumulation of GABA_A_Rs [46]–[50], the severe phenotype and reduced life span of γ2 KD mice are likely due to a strong decrease of synaptic GABAergic transmission (see below). We did not examine why the cDNA insertion disrupted γ2 gene expression; other than the original report mapping the γ2 gene's transcriptional start sites and proximal promoter [20], nothing more has become known about how this gene is regulated. Nevertheless, although we could not use the mice for their intended purpose of allowing Cre-inducible zolpidem sensitivity in particular brain regions [44], the line did offer an excellent opportunity to study the role of GABA in postnatal brain development and synaptogenesis.

### Normal brain assembly in γ2 KD mice

**Figure 3:**
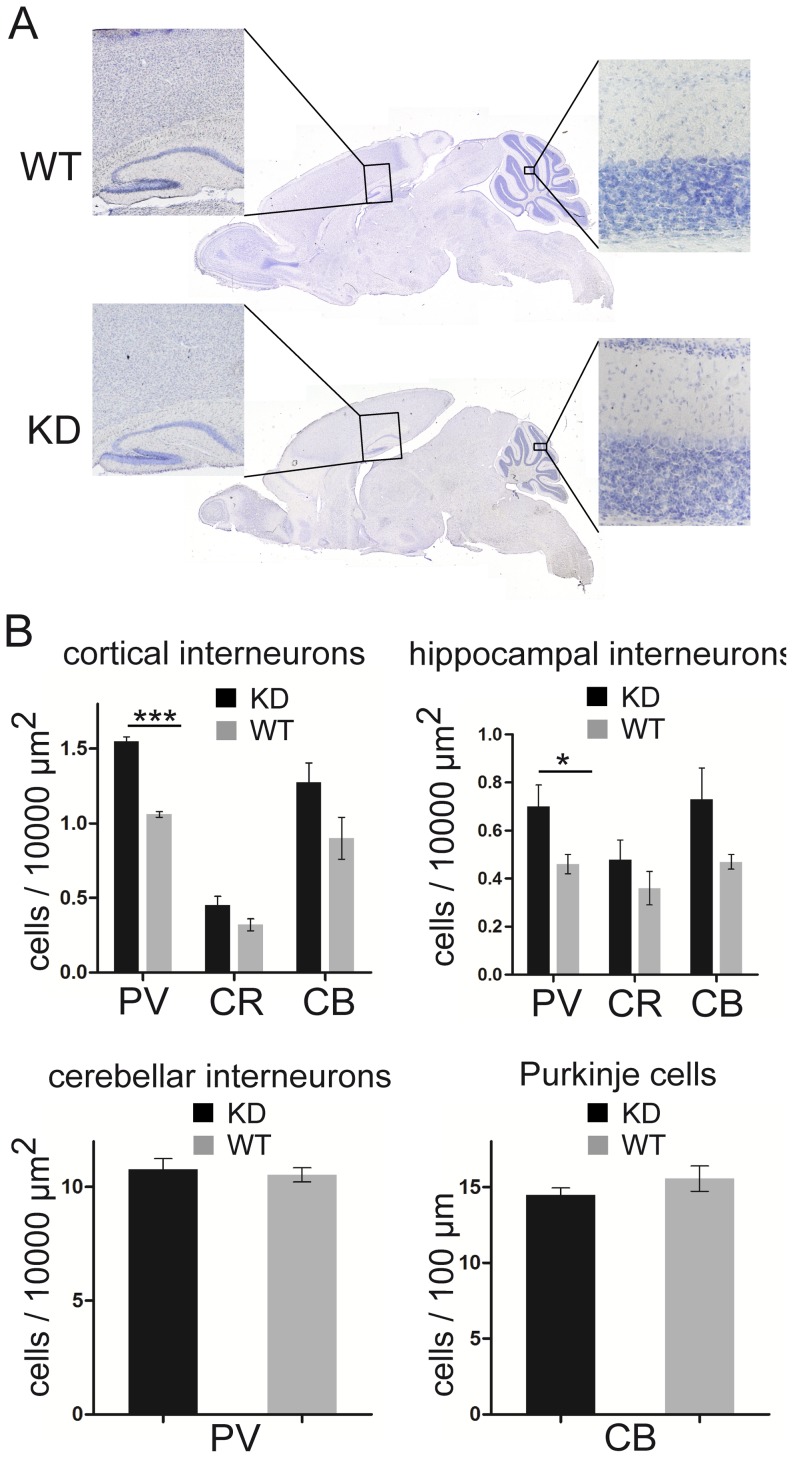
Normal brain architecture in γ2 KD mice. **A,** Nissl staining reveals a similar morphology in the brain of a γ2 KD mouse and a WT littermate. **B,** Quantitative analysis based on immunofluorescence labeling using antibodies against calcium binding proteins. The density of parvalbumin (PV)-positive cells in the hippocampal CA1 and sensorimotor cortex of γ2 KD mice was significantly increased with respect to WT (***, p<0.0001; *, p = 0.0352; unpaired *t*-test; n = 4 mice per group). The density of MLIs and PCs in the cerebellum was unaltered (MLIs, p = 0.6653; PCs, p = 0.3224; unpaired *t*-test; n = 5 mice per group). CB, calbindin; CR, calretinin.

The brains of γ2 KD mice seemed correctly assembled. Nissl staining revealed that the brains of mutant and control littermates were morphologically similar, although γ2 KD brains were slightly smaller, consistent with the reduced size of mutant mice (Fig. 3A). In particular, cortical layering appeared normal in γ2 KD mice, as also supported by normal expression of neocortical lamination markers seen by in situ hybridization (not shown). In addition, no sign of heterotopia or neuronal cysts were visible in the cerebral and cerebellar cortices. Using antibodies against nonoverlapping subtypes of GABAergic interneurons, we found a general trend towards increased densities of interneurons in the hippocampus and sensorimotor cortex of γ2 KD mice, which however reached significance only for the subgroup of parvalbumin-positive neurons (Fig. 3B). In the cerebellar cortex, double labeling for parvalbumin and calbindin revealed a normal density of PCs and molecular layer interneurons (Fig. 3B). These data indicate that reduced signaling through γ2-containing GABA_A_Rs has no major effects on neuronal differentiation and interneuron survival. The increased density of parvalbumin-positive interneurons in cortical areas may be a compensatory mechanism to counteract the strong decrease of GABAergic inhibition.

### Mosaic expression of the γ2 subunit in neurons of γ2 KD mice

**Figure 4:**
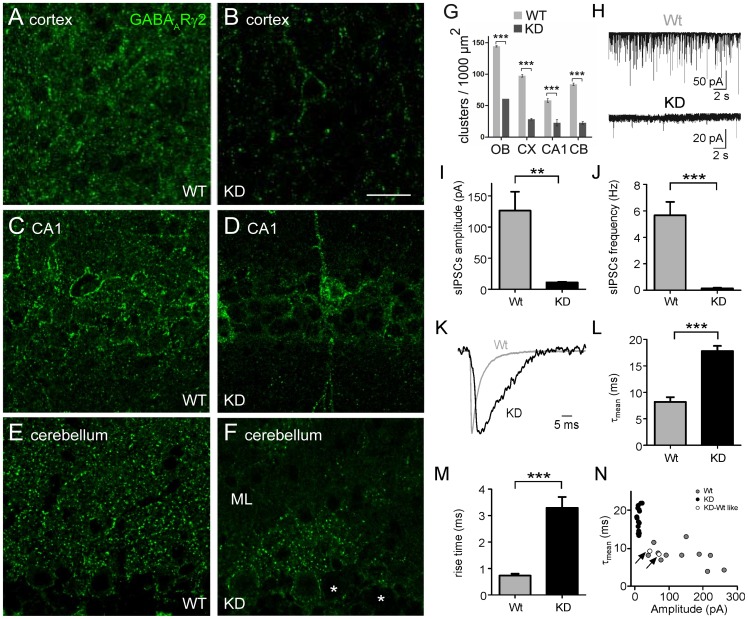
Mosaic expression of the γ2 subunit in the brain of γ2 KD mice. **A–F,** Representative images of sensorimotor cortex, hippocampal CA1 and cerebellum showing immunofluorescence labeling for the γ2 subunit. Note the reduced punctate labeling in γ2 KD brains as compared with WT. In γ2 KD cerebellum the co-existence of γ2-positive and γ2-negative PCs (asterisks) results in an uneven distribution of synaptic clusters in the molecular layer (ML). **G,** Quantification of γ2-positive puncta in brain regions of γ2 KD mice and WT littermates (***, p<0.001; unpaired *t*-test; n = 3 mice per group). OB, olfactory bulb (external plexiform layer); CX, sensorimotor cortex (layer V); CA1, CA1 (*stratum radiatum*); CB, cerebellum (molecular layer). **H,** Example traces of sIPSCs recorded from PCs in WT and γ2 KD mice. **I,** Quantitative analysis showing dramatically reduced amplitude of sIPSCs recorded from γ2 KD PCs (n = 17 cells) compared with WT (n = 11 cells; **, p = 0.0066; unpaired *t*-test). **J,** Reduced frequency of sIPSCs recorded from γ2 KD PCs (n = 17 cells) compared with WT (n = 11 cells; ***, p<0.0001; unpaired *t*-test) **K,** Normalized and superimposed representative traces of sIPSCs recorded from WT (gray) and KD cells (black). **L,** Quantification of current deactivation (τ_mean_) in WT (n = 11) and KD cells (n = 17). **M,** Current onset kinetics (10–90% rise time) of sIPSCs from WT and γ2 KD neurons. **N,** Distributions of deactivation (τ_mean_) and amplitude of sIPSCs recorded from PCs in WT and γ2 KD slices. Each data point represents the τ_mean_ and amplitude values of individual recordings. Arrows indicate the values of two PCs recorded from γ2 KD slices showing WT-like current properties. Data represent mean ± SEM (***, p<0.001; unpaired *t*-test). Scale bar: 25 µm.

**Figure 5:**
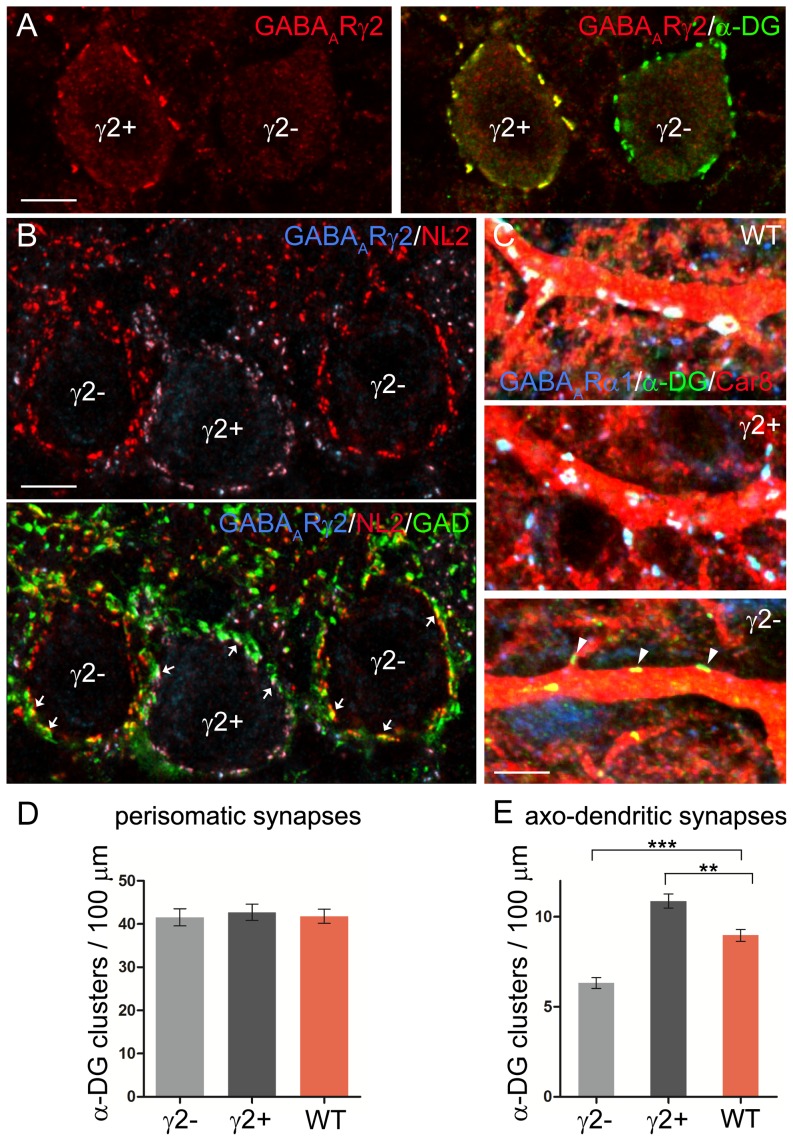
Postsynaptic GABA_A_Rs determine axo-dendritic but not perisomatic innervations patterns in cerebellar PCs. **A,** Perisomatic synapses in PCs of a γ2 KD mouse. α-DG (green) co-localizes precisely with the γ2 subunit (red) in a γ2-positive PC (γ2+) and also outlines the profile of a γ2-negative PC (γ2−). **B,** Upper panel: NL2 (red) co-localizes precisely with the γ2 subunit (blue) in a γ2-positive PC (γ2+) and also outlines the profile of two γ2-negative PCs (γ2−). Lower panel: triple labeling shows that NL2 clusters opposite GAD65-positive boutons (green) in both γ2-positive and γ2-negative PCs (arrows). **C,** Confocal images of PC dendritic profiles after triple labeling for GABA_A_R α1 (blue), α-DG (green) and Car8 (red). DG co-localizes with GABA_A_R clusters in PCs of WT mice as well as in γ2-positive PCs of γ2 KD mice (the superposition of the three fluorescent channels results in white clusters). The lower panel shows a γ2-negative dendritic profile, where α-DG clusters are not associated with GABA_A_Rs (triangles). **D,** The density of perisomatic synapses is similar in γ2-positive, γ2-negative and WT PCs (γ2+ *vs* γ2−, p = 0.6920; γ2+ *vs* WT, p = 0.7312; γ2− *vs* WT, p = 0.9230; unpaired *t*-test; n = 5 mice per group). **E,** The density of α-DG clusters is lower in γ2-negative dendrites and higher in γ2-positive dendrites compared with the WT situation (**, p = 0.0064, ***, p = 0.0003; unpaired *t*-test; n = 5 mice per group). Scale bars: A,B = 10 µm. C = 5 µm.

We used immunofluorescence to investigate the distribution of the γ2 subunit in brains of γ2 KD mice. Labeling for the γ2 subunit was punctate, suggesting synaptic localization (Fig. 4A–F). However, in all regions analyzed there was a noticeable reduction in the density of γ2-positive puncta as compared with the WT situation (Fig. 4G). Remarkably, the γ2 subunit appeared to have a mosaic expression, resulting in the presence of γ2-positive and γ2-negative neurons co-existing in the same areas. This was particularly evident in the cerebellar cortex, where more than 60% of PCs were γ2-negative, as determined by the absence of immunolabeled puncta outlining the cell body (Fig. 4F). Notably, the percentage of γ2-negative PCs was constant from P7 (64%, *n* = 67 cells), when perisomatic synapses are initially assembled, to P20 (63%, *n* = 127 cells), suggesting that the majority of PCs do not express the γ2 subunit during the entire period of postnatal development. Moreover, the reduced expression of the γ2 subunit was paralleled by a similar decrease of puncta immunolabeled for the α1 subunit (Fig. 5C), indicating that loss of the γ2 subunit caused a disruption of postsynaptic GABA_A_Rs.

Patch-clamp recordings were performed on PCs to ascertain how downregulation of the γ2 subunit affects GABAergic synaptic transmission. We recorded spontaneous inhibitory postsynaptic currents (sIPSCs) from cerebellar acute slices of γ2 KD and WT mice aged P15–P18. The majority of PCs exhibited a markedly reduced synaptic activity as compared with WT cells (Fig. 4H). Indeed, we observed a strong reduction in sIPSC amplitude from 136.3±30.3 pA (n = 11) in WT mice to 10.9±1.2 pA (n = 17) in KD cells (Fig. 4I). Similarly, a strong reduction in the frequency of sIPSCs was observed in KD neurons (WT: 5.25±1.00 Hz; KD: 0.15±0.04 Hz; Fig. 4J). An additional analysis aimed at studying the kinetic properties of synaptic currents upon knock down of the γ2 subunit revealed that sIPSCs recorded from KD cells exhibited slower deactivation and onset (Fig. 4K). The τ_mean_ of current deactivation increased from 8.2±0.9 ms in WT cells to 17.8±1.0 ms in KD cells (Fig. 4L) and, similarly, the 10–90% sIPSC rise time increased from 0.73±0.06 ms in WT to 3.30±0.41 ms in KD cells (Fig. 4M). Notably, two PCs recorded from γ2 KD slices had sIPSC properties (amplitude: 56.5±13.4 pA; frequency: 3.1±0.17 Hz; τ_mean_: 8.902±0.41 ms) that approached those of WT, suggesting that they contained γ2-positive clusters (Fig. 4N). Therefore, loss of the γ2 subunit causes a dramatic decrease of inhibitory postsynaptic currents in PCs, and the few remaining currents have considerably slower kinetics compared with the WT situation.

### Synaptic competition determines axo-dendritic innervation patterns in cerebellar PCs

The data so far indicate that mosaic expression of the γ2 subunit causes a strong imbalance in GABAergic activity in neighboring neurons. This situation is ideal for studying the importance of GABAergic signaling for synapse development. We have shown previously that deletion of the GABA_A_R α1 subunit does not affect the postsynaptic localization of NL2 and α-DG in PCs [18]. Similarly, we found that NL2 and α-DG clustered at postsynaptic sites facing GAD65-positive boutons in PCs lacking the γ2 subunit (Fig. 5A–C). We estimated the density of NL2 and GAD65-positive structures in the molecular layer of γ2 KD mice. Compared to WT, NL2 puncta and GAD65 terminals were decreased respectively by 34% (mean ± SEM puncta/1000 µm^2^: 79.9±2.9 in WT; 52.7±4 in KD; p = 0.0012; unpaired t-test; n = 3 mice per group) and 36% (mean ± SEM puncta/1000 µm^2^: 68.5±1.6 in WT; 44±1.4 in KD; p = 0.0001; unpaired *t*-test; n = 3 mice per group), indicating that knockdown of the γ2 subunit results in a similar decrease of the density of pre- and postsynaptic structures. We then used antibodies against NL2 and/or α-DG to compare synapse organization in neighboring PCs that were either γ2-positive or γ2-negative. The results were compared with the situation in WT littermates. The density of perisomatic synapses was similar in γ2-positive, γ2-negative and WT PCs (Fig. 5D), substantiating the idea that the development of perisomatic synapses in PCs does not depend on GABAergic activity levels [17]. We then analyzed axo-dendritic synapses, using antibodies against Car8 to label selectively PC dendrites (Fig. 5C). Given that the development of axo-dendritic synapses is influenced by GABAergic signaling [17], [18], our prediction was that the strong imbalance in the expression of the γ2 subunit would cause a reduction of GABAergic innervation in γ2-negative PCs, as well as increased connectivity in γ2-positive PCs. Indeed, the density of DG-positive synapses was markedly reduced in γ2-negative dendrites. Conversely, synapse density was significantly higher in γ2-positive dendrites compared with both γ2-negative and WT PCs (Fig. 5E). These data suggest that the number of axo-dendritic synapses that are established during development is strongly influenced by the level of GABAergic activity in the postsynaptic neurons.

### Mosaic expression of the γ2 subunit prevents the formation of heterologous synapses in γ2 KD cerebella

**Figure 6:**
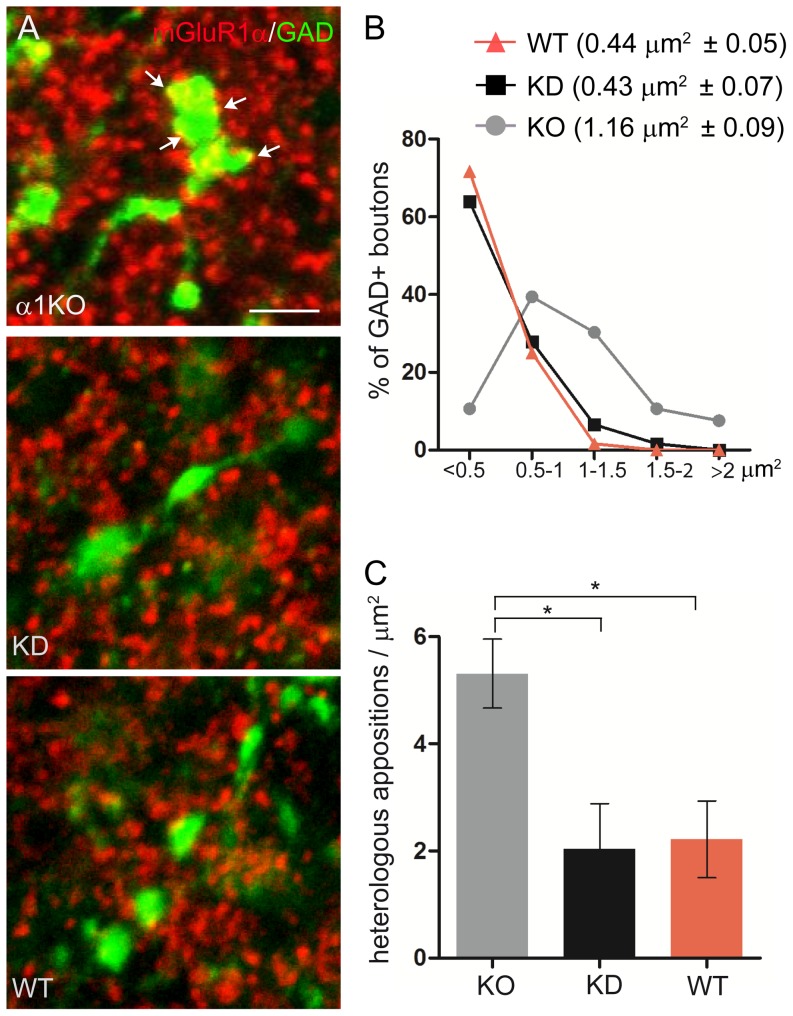
Absence of heterologous synapses in the cerebellum of γ2 KD mice. **A,** Representative confocal images after double labeling for mGluR1α (a marker of PC spines) and GAD65. In global α1 knockout mice (upper panel), large GABAergic axon terminals make multiple contacts with PC spines (arrows). In γ2 KD (middle panel) and WT mice (lower panel), GAD65-positive terminals have a smaller size and are less frequently found in close apposition with spines. **B,** Distribution of GAD65-positive boutons in the molecular layer of total α1 knockouts, γ2 KD and WT mice based on size. The average area (± SEM) is indicated. In total α1 knockout mice, GABAergic axon terminals are significantly larger than in the other groups (α1 KO *vs* WT, p = 0.0016; α1 KO *vs* γ2 KD, p = 0.0008; γ2 KD *vs* WT, p = 0.9348; unpaired *t*-test; n = 4 mice per group). **C,** The density of heterologous contacts between GAD65-positive terminals and PC spines is significantly higher in global α1 knockout mice compared with both γ2 KD and WT mice (α1 KO *vs* WT, p = 0.03; α1 KO *vs* γ2 KD, p = 0.03; γ2 KD *vs* WT, p = 0.8836; unpaired *t*-test; n = 3 mice per group). Scale bar: 2 µm.

Mouse models that lack GABAergic transmission in all PCs (global α1 knockout mice [17], [18]; PC-Δα1 mice [51]; PC-Δγ2 mice [49]) are characterized by reduced axo-dendritic innervation and the presence of heterologous synapses in which several PC spines are contacted by an unusually large GABAergic terminal. To see if in γ2 KD mice GABAergic axons are similarly attracted by improper postsynaptic targets, we performed double labeling for GAD65 to identify GABAergic terminals and mGluR1α to label PC spines (Fig. 6A; see also ref. [18]), and made a direct comparison with global α1 knockouts. In γ2 KD mice GABAergic boutons were significantly smaller than in global α1 knockouts, being close in size to those of WT animals (Fig. 6B). Moreover, in both γ2 KD and WT littermates GABAergic terminals were rarely found in close apposition with spines, whereas in α1 knockouts the number of heterologous contacts was significantly higher (Fig. 6C). These data suggest that heterologous synapses are an aberrant phenotype that occurs in situations in which *all* PCs are rendered silent to GABAergic transmission.

### Target zone-specific differences in GABAergic synapse development are not restricted to the cerebellar cortex

**Figure 7:**
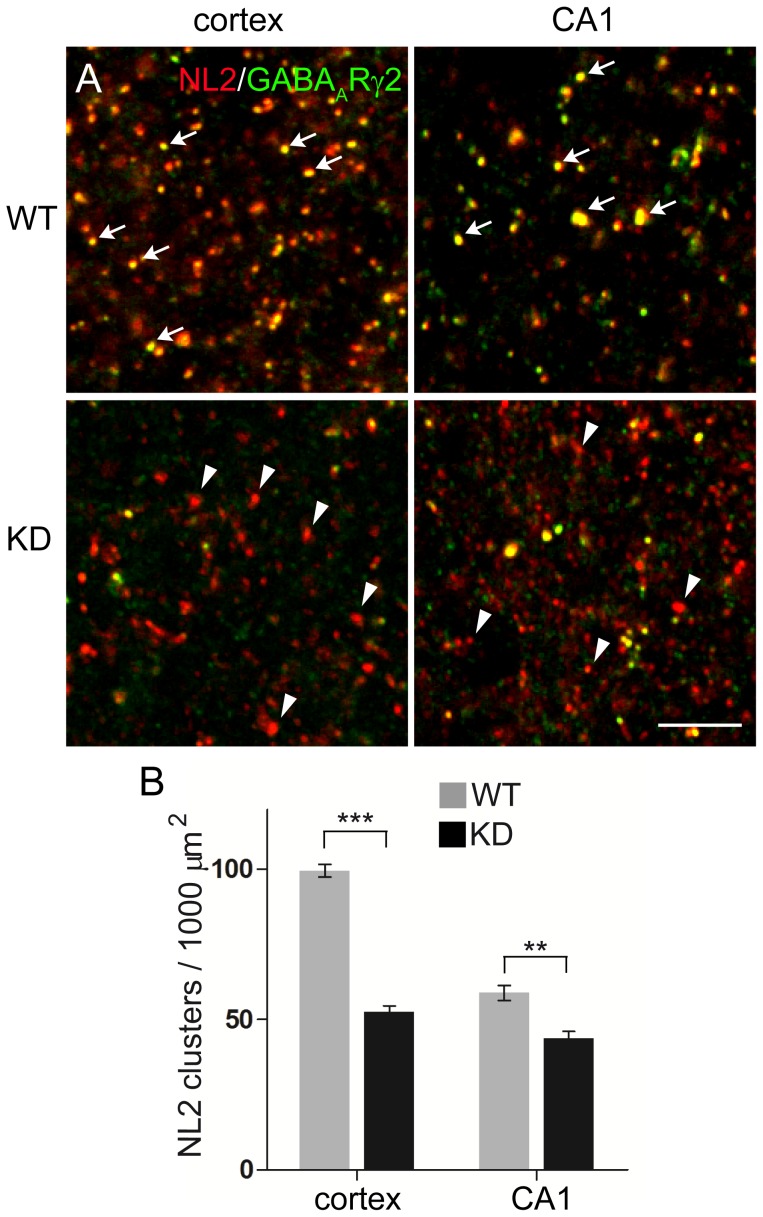
NL2 clusters at synapses lacking γ2-GABA_A_Rs in cortical and hippocampal circuits. **A,** Confocal images of sensorimotor cortex (layer V) and hippocampal CA1 (*stratum radiatum*) after double labeling for NL2 (red) and GABA_A_R γ2 (green). Note that in WT NL2 puncta co-localize extensively with γ2-positive structures (arrows). In contrast, in γ2 KD mice many puncta are labeled for NL2 but not for the γ2 subunit (triangles). **B,** Reduced density of NL2-positive clusters in cortical and hippocampal neuropil of γ2 KD mice as compared with WT (***, p<0.0001,** p = 0.0031; unpaired *t*-test; n = 3 mice per group). Scale bar: 3 µm.

**Figure 8:**
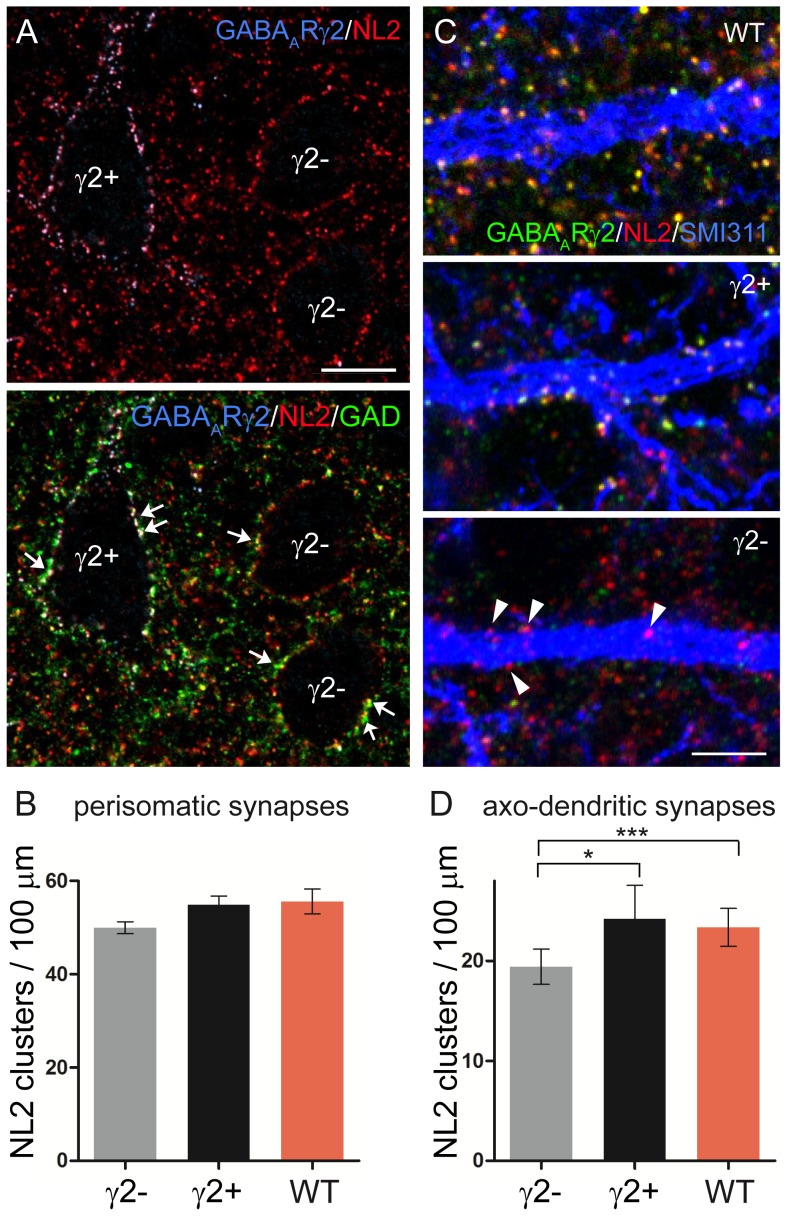
Perisomatic and axo-dendritic synapses of pyramidal cortical neurons have different dependencies on synaptic GABA_A_Rs. **A,** NL2 (red) co-localizes precisely with the γ2 subunit (blue) in a γ2-positive pyramidal neuron (γ2+) and also outlines the profile of two γ2-negative cells (γ2−) in layer V of sensorimotor cortex of a γ2 KD mouse (upper panel). Lower panel: triple labeling shows that NL2 clusters opposite GAD65-positive boutons (green) in both γ2-positive and γ2-negative pyramidal cells (arrows). **B,** The density of perisomatic synapses is similar in γ2-positive, γ2-negative and WT pyramidal neurons (γ2− *vs* WT, p = 0.0748; γ2+ *vs* WT, p = 0.8187; γ2+ *vs* γ2−, p = 0.0602 unpaired *t*-test; n = 3 mice per group). **C,** Confocal images of dendritic profiles after triple labeling for GABA_A_R γ2 (green), NL2 (red) and SMI 311 (blue). NL2 co-localizes with GABA_A_R clusters in pyramidal neurons of WT mice as well as in γ2-positive pyramidal neurons of γ2 KD mice. The lower panel shows a γ2-negative dendritic profile, where NL2 clusters are not associated with GABA_A_Rs (triangles). **D,** The density of NL2 clusters is significantly lower in γ2-negative dendrites compared with the other two groups, whereas no difference was found between γ2-positive and WT dendrites (γ2− *vs* WT, p = 0.0008; γ2− *vs* γ2+, p = 0.04; γ2+ *vs* WT, p = 0.83; unpaired *t*-test; n = 3 mice per group). Scale bars: A = 10 µm. C = 3 µm.

The results obtained in PCs indicate that competition mediated by synaptic GABA_A_Rs sculpts the development of axo-dendritic, but not perisomatic inhibitory synapses. To understand whether this is a general principle in GABAergic synapse development, we extended our analysis to other neuronal circuits. We initially asked whether the absence of the γ2 subunit affects postsynaptic clustering of NL2 in different types of neuron and different types of GABAergic synapse. We frequently observed NL2-positive, γ2-negative clusters in the hippocampus and neocortex (Fig. 7A), as well as in several other brain regions (not shown). These punctate structures were present in the neuropil and also around cell bodies. Triple labeling for the γ2 subunit, NL2 and GAD65 revealed that NL2 clustered at presumed GABAergic synapses lacking postsynaptic GABA_A_Rs (Fig. 8A). These data extend our previous observations in PCs [18] and indicate that GABA_A_Rs are not required for postsynaptic accumulation of NL2 at perisomatic and axo-dendritic GABAergic synapses. Interestingly, γ2 KD mice had a lower density of NL2-positive clusters in synaptic layers in both CA1 (*stratum radiatum*) and sensorimotor cortex (layer V; Fig. 7B). This is reminiscent of the situation in the cerebellum (see above), and suggests that silencing of synaptic GABAergic transmission decreases the number of GABAergic synapses in dendritic domains (see below).

We then analyzed the organization of perisomatic and axo-dendritic synapses in pyramidal neurons of sensorimotor cortex layer V. This region was selected because γ2-positive and γ2-negative pyramidal cells were clearly discernible using antibodies against the γ2 subunit and NL2 (Fig. 8A). We found no significant differences in the density of NL2 clusters outlining the profile of γ2-positive, γ2-negative and WT pyramidal cells, suggesting that perisomatic innervation of cortical neurons is not regulated by synaptic GABA_A_Rs (Fig. 8B). We then quantified synapse density along the dendrites of pyramidal neurons labeled with an antibody against SMI 311 (Fig. 8C). In γ2-negative dendrites there was a significant decrease in the density of NL2-positive clusters compared with both γ2-positive and WT dendrites (Fig. 8D). Dendrites that were positive for the γ2 subunit had a slightly elevated density of GABAergic contacts compared with the WT situation, however this difference was not significant (Fig. 8D). It should be noted, however, that labeling for SMI 311 did not fill the entire dendritic arborization of pyramidal neurons, therefore our analysis was restricted to the more proximal dendritic domains, where synapse number appears to be less influenced by activity levels [18]. These data indicate that in neocortical neurons, like in PCs, perisomatic and axo-dendritic synapses have different dependencies on GABAergic transmission.

## Discussion

To investigate the importance of GABAergic signaling in synapse development, we have taken advantage of an engineered *gabrg*2 mouse allele that strongly reduced the expression of synaptic GABA_A_Rs. The γ2 subunit is essential for postsynaptic aggregation of GABA_A_Rs [46], and its deletion dramatically affects inhibitory postsynaptic currents (refs. [44], [47]–[49] and Fig. 4H–J). Global ablation of the γ2 subunit in mice causes perinatal lethality [19], [46], thus preventing *in vivo* analyses of synapse differentiation. In contrast, the γ2 KD mice reported here survive until their third postnatal week, when synaptogenesis has reached an advanced stage in most brain regions, making these mice useful to study GABA's developmental role during the peak period of synaptogenesis.

### Knockdown of the γ2 subunit strongly downregulates GABAergic synaptic currents

The strongly reduced GABAergic synaptic activity observed in γ2 KD mice is consistent with previous studies in which the γ2 subunit was deleted in neurons by Cre-mediated recombination [44], [48], [49]. In both cases, the residual sIPSCs had small peak amplitudes and slow decay time constants. This effect is most likely due to spillover of synaptically released GABA onto low-conductance α/β GABA_A_Rs [52]. Given the key role of the γ2 subunit for the synaptic localization of GABA_A_Rs [46], [53], it can be speculated that in γ2 KD cells the activation of α/β-containing receptors dispersed in the perisynaptic and extrasynaptic membrane would be delayed by the time needed for GABA to diffuse outside the synaptic cleft. As a consequence, the macroscopic sIPSC onset and decay kinetics would be delayed. It cannot be excluded that loss of the γ2 subunit also affects the gating properties of GABA_A_Rs [54], with a direct impact on synaptic current kinetics.

A few PCs in γ2 KD slices had synaptic currents similar to those of WT, as predicted by the co-existence of γ2-positive and γ2-negative cells revealed by immunofluorescence (Fig. 4N). These cells had somewhat lower amplitudes and frequencies compared to the average WT values, suggesting that even γ2-positive PCs may express lower-than-normal levels of the γ2 subunit. Verification of this assumption would require data from a larger population of PCs, which has been hampered so far due to the limited availability of mutant mice. However, our data clearly demonstrate that expression of the γ2 subunit was sufficient to rescue the deficit in the formation of axo-dendritic synapses in γ2-positive PCs (see below).

### Knockdown of the γ2 subunit has no major effects on brain development

Surprisingly, impaired GABA signaling in γ2 KD mice did not interfere with normal brain assembly and cortical lamination. This might appear in contrast with many studies indicating that GABA is an important regulator of cell proliferation, neuroblast migration and neuronal differentiation [55]–[62]. However, the residual expression of α/β GABA_A_Rs in neurons lacking the γ2 subunit (see above) leaves open the possibility that GABA might exert nonsynaptic effects. On the other hand, the absence of major neurodevelopmental defects in γ2 KD mice is consistent with other investigations that have revealed a largely normal brain architecture in mice with null mutations in key genes of the GABA pathway [63]–[66]. It remains possible that γ2 KD brains present subtle defects. For example, the increase in the population of parvalbumin-positive interneurons that we observed in the hippocampal CA1 and sensorimotor cortex (Fig. 3B) may influence the function and plasticity of cortical circuits [67]–[70].

### γ2-GABA_A_Rs are not essential for postsynaptic clustering of NL2

An important goal of our study was to determine how γ2-GABA_A_Rs regulate the developmental assembly of GABAergic synapses. Ideally, to visualize synapses by immunohistochemistry, one should co-stain sections with antibodies directed against both pre- and postsynaptic markers (Fig. 5B, 8A). To distinguish between γ2-positive and γ2-negative neurons, however, we were forced to use a brief-fixation protocol that has been optimized for the detection of postsynaptic molecules [18], [42]. Therefore, quantification of GABAergic synapses was mainly based on labeling for NL2 and/or α-DG as markers of the postsynaptic specialization.

The γ2 subunit is a crucial organizer of GABAergic synapses and may stabilize postsynaptic receptor aggregates by directly interacting with other transmembrane proteins [50], [53], [71]. One hypothesis is that the developmental assembly of GABAergic synapses depends on an activity-dependent link between GABA_A_Rs and NL2, although it is unclear whether these proteins interact directly [15], [72]. Previous studies have indicated that NL2 clusters at postsynaptic sites lacking GABA_A_Rs in PCs of mutant mice [18], [51]. Similarly, we found here that NL2 clusters faced GAD65-labeled boutons in neurons lacking γ2-containing GABA_A_Rs (Fig. 5B, 8A and data not shown), indicating that GABA_A_Rs are not essential for recruiting NL2 to the postsynaptic specialization. A recent study, however, found that clustering of NL2 at axo-axonic synapses of CA1 hippocampal neurons largely depends on α2-containing GABA_A_Rs [73], suggesting that NL2-GABA_A_R interactions may be synapse-specific. On the other hand, studies in NL2 knockout mice have indicated that NL2 contributes to stabilize postsynaptic GABA_A_Rs, at least in specific types of inhibitory synapses, and is required for normal GABAergic transmission [72], [74]–[77]. Collectively, the data indicate that neither NL2 nor GABA_A_Rs are essential for the formation of morphologically-recognizable inhibitory synapses; however, NL2 and GABA_A_Rs interact in a synapse-specific manner to organize postsynaptic specializations and determine synaptic properties [50], [78]. One mechanism by which NL2 seems to regulate the maturation of GABAergic synapses is a direct interaction with gephyrin and collybistin, which promotes the formation of a postsynaptic scaffold onto which GABA_A_Rs are tethered [76].

### Different regulation of perisomatic and axo-dendritic synapses

In all regions of the γ2 KD brain analyzed, there was a decrease in the density of NL2-positive puncta in synaptic layers (Fig. 7), suggesting that silencing of GABAergic transmission perturbs axo-dendritic synapse development. This was confirmed by high-resolution analyses on the dendrites of cerebellar PCs (Fig. 5C,E) and cortical pyramidal neurons (Fig. 8C,D), that revealed a decreased density of GABAergic postsynaptic structures in neurons lacking the γ2 subunit. These data are consistent with previous studies on cultured neurons that demonstrated that γ2-subunit containing GABA_A_Rs are essential for normal GABAergic innervation [79], [80]. However, we found that the number of perisomatic postsynapses was not affected by loss of synaptic GABA_A_Rs, providing strong support to the idea that perisomatic and axo-dendritic synapses have different dependencies on GABAergic activity levels. Li et al. (ref. [79]) reported a modest reduction of perisomatic innervation (24–29% compared with a 53% reduction of GABA_A_R cluster density) of cortical neurons after in utero electroporation of γ2 shRNAs. The slight discrepancy between our results (no significant effect on perisomatic synapses) and those of Li et al. (modest reduction of perisomatic innervation) could be possibly explained by differences in the sensitivity of the immunolabeling procedure, or by differences in the quantification method, that was based on immunolabeling for the presynaptic vesicular GABA transporter (VGAT) in Li et al. [79] and on labeling for NL2 in the present investigation. In support of our observations, a recent study [73] has also shown that deletion of α2-GABA_A_Rs does not affect perisomatic innervation in CA1 pyramidal neurons, although compensation by the α1 subunit has to be taken into account in this case.

In contrast with the fixed situation of perisomatic synapses, the development of axo-dendritic synapses was sensitive to differences of GABAergic activity among neighboring cells. Our data clearly demonstrate that PCs lacking the γ2 subunit are disadvantaged for axo-dendritic synapse formation or stabilization, whereas their neighbors expressing the γ2 subunit increased the number of inhibitory postsynaptic sites compared with WT PCs. A similar situation has been reported in cultured neurons, where knockdown of the γ2 subunit [79] or the palmitoyltransferase GODZ [80] caused a disruption of GABA_A_R clusters and selectively impaired GABAergic innervation.

Most likely, a similar activity-dependent process was responsible for the absence of heterologous synapses on spines in γ2 KD mice. Heterologous synapses are abundant in situations in which there is a uniform suppression of GABAergic transmission in all PCs [17], [18], [51]. However, there is probably no advantage in maintaining heterologous synapses in the competitive environment of the γ2 KD cerebellum. In other words, expression of the γ2 subunit in at least some PCs resulted in increased axo-dendritic connectivity and was sufficient to avoid the formation and/or maintenance of heterologous synapses. These observations also indicate that axo-dendritic innervation does not depend on a hard-wired process based on exclusive molecular interactions, but results from a mechanism of selection among potential synaptic partners. In normal conditions, GABA signaling serves to determine the density of synapses within the dendritic arborization of individual PCs. When the preferred connections are silenced, however, synapses can form with alternative partners, including dendritic spines.

The differences between perisomatic and axo-dendritic synapses could be related to their different roles in neuronal networks. Perisomatic synapses are known to control neuronal output very efficiently and are involved in neuronal synchronization [69], [81]–[83]. This requires that their number is strictly determined during development, and scarcely influenced by activity levels. Conversely, synapses on dendrites regulate glutamatergic inputs and calcium signals and exhibit a higher degree of activity-dependent plasticity [84]–[86]. Under this assumption, the level of plasticity at maturity is the main determinant of the relative importance of activity versus molecular cues in the assembly of inhibitory synapses during development.

The downstream pathways linking postsynaptic GABA_A_Rs to synapse maturation are presently unclear. Obviously, loss of GABA_A_Rs not only affects inhibitory neurotransmission, but also impairs molecular interactions within synaptic complexes. However another study has demonstrated that knockdown of GABA synthesis in cortical interneurons inhibits the ability of GABAergic axons to establish synapses with the appropriate targets, supporting the idea that GABAergic synapses are stabilized by an activity-dependent mechanism [16]. During cerebellar development, GABAergic synapses made by molecular layer interneurons are characterized by presynaptic miniature currents (preminis) that depend on the activation of presynaptic GABA_A_Rs and enhance neurotransmitter release [87]. The authors have proposed that depolarization due to preminis and autoreceptor activation produces a feedback loop that maintains a high release probability at recently formed synapses. Combined with our present findings, this suggests that axodendritic synapses could be stabilized by a mechanism involving the combined activation of GABA_A_Rs located at both pre- and postsynaptic sites. This hypothesis could be tested by analyzing how a selective ablation of GABA_A_Rs from molecular layer interneurons affects synapse development.

In conclusion, our findings reveal a remarkable selectivity in the way that synaptic activity determines the stoichiometry of synaptic connections in distinct subcellular compartments. Interestingly, the resilience of perisomatic synapses observed during development matches the situation in the aging brain, when synapses located on dendritic domains are significantly reduced while those located on the cell body are relatively unaffected [88]. Similarly, perisomatic synapses are spared, if not potentiated, in some forms of intractable epilepsies, and may contribute to the generation of pathological network activity [89], [90]. Thus, understanding the different dependency of perisomatic and dendritic synapses on activity levels may be relevant for deciphering brain disorders that arise from altered GABAergic activity or changes in the excitatory/inhibitory balance.
